# Hyphal penetration is the major pathway of translocation of *Candida albicans* across the blood-cerebrospinal fluid barrier

**DOI:** 10.1186/s12987-025-00644-x

**Published:** 2025-04-04

**Authors:** S. Schmidt, C. Schwerk, H. Schroten, H. Ishikawa, R. Schubert, T. Lehrnbecher, H. Rudolph

**Affiliations:** 1 Department of Pediatrics, Division of Hematology, Oncology and Hemostaseology, Goethe University Frankfurt, Frankfurt/Main, Germany; 2 Pediatric Infectious Diseases, Medical Faculty Mannheim, University Children’s Hospital Mannheim, Heidelberg University, Mannheim, Germany; 3 Laboratory of Regenerative Medicine, Department of Neurosurgery, Faculty of Medicine, University of Tsukuba, Tsukuba, Japan; 4 Department of Pediatrics, Division of Pneumology, Allergology, Infectious Diseases and Gastroenterology, Goethe University Frankfurt, Frankfurt/Main, Germany

**Keywords:** Blood cerebrospinal fluid barrier, Choroid plexus, *C. albicans*, Hyphal penetration, Tight junctions

## Abstract

**Background:**

Despite the availability of potent antifungal compounds, invasive fungal disease poses significant morbidity and mortality in immunocompromised patients. *Candida albicans* is one of the leading pathogens in this setting, and may affect the central nervous system (CNS), which is an extremely severe form of the infection. As the exact pathogenesis of *Candida* CNS infection is not clear, we investigated the mechanisms and effects of *C. albicans* transmigration into the CNS, which will be helpful for diagnosis, prevention and treatment.

**Methods:**

We used a human in vitro model of the Blood-Cerebrospinal Fluid Barrier (BCSFB), and we investigated the mechanisms of *Candida albicans* translocation into the CNS. Translocation was evaluated using immunofluorescence analysis focusing on tight and adherens junctions and the actin cytoskeleton. Barrier integrity was monitored via measurement of transepithelial resistance and the paracellular permeability of dextran. LIVE/DEAD assays were applied for viability controls and a cytometric bead array was performed to detect cytokine secretion of plexus epithelial cells.

**Results:**

Translocation at low doses occurs transcellularly in the absence of cytotoxicity or secretion of proinflammatory cytokines. This is accomplished by the formation of a tunnel-like structure exploiting the actin cytoskeleton. With higher infection doses of *Candida albicans*, a reduction in barrier integrity due to disruption of tight and adherens junctions was observed and cytotoxicity also increased.

**Conclusion:**

Our findings reveal that *Candida albicans* can use transcellular translocation to invade into the CNS and is able to circumvent major host immune response, which may impact on diagnostic and preventive strategies.

**Supplementary Information:**

The online version contains supplementary material available at 10.1186/s12987-025-00644-x.

## Background

Despite major advances in prophylaxis and treatment, invasive fungal disease (IFD) is a major cause of morbidity and mortality in immunocompromised patients, such as those receiving cancer treatment, undergoing hematopoietic or organ transplantation, or suffering from HIV/AIDS [1]. Autopsy studies demonstrated a significant increase of IFD over the last decades, with *Aspergillus spp.* and *Candida spp.* as the leading pathogens [2–4]. The importance of IFD in the treatment of immunocompromised patients is underlined by a recent study reporting that IFD is an independent risk factor for poor outcome in children treated for acute lymphoblastic leukemia (ALL) [5].

*Candida albicans* is an opportunistic pathogen and can be found in most healthy individuals in the oral, genital and intestinal mucosa [6]. Although the mortality of invasive *Candida* infections is lower compared to that caused by molds, *Candida* spp. may cause severe disseminated infection affecting organs such as liver, spleen or the central nervous system (CNS) [7]. Robust epidemiological data on the disease burden of CNS infections caused by fungi are not available, but the incidence of CNS-IFD seems to increase [8]. Fungal infections of the CNS usually originate from primary sites outside the CNS (e.g. fungal pneumonia) and represent an extremely severe form of infection that is challenging to diagnose and to treat, and often result in fatal outcome or permanent neurological sequelae [8, 9]. In changing environments within the host, *C. albicans* grows in different forms, namely yeast cells, pseudohyphae and hyphae. Hyphae develop from ungerminated yeast cells and are described to be majorly involved in invasion of the gut epithelium [10, 11].

In order to improve both diagnostic and therapeutic approaches in CNS-IFD, it is essential to understand the exact pathogenesis of the infection. To invade the CNS, *Candida* spp. has to overcome either the Blood-Brain-Barrier (BBB) or the Blood-Cerebrospinal Fluid-Barrier (BCSFB), which may occur by induced endocytosis, transcellular translocation, paracellular translocation via breaching tight and adherens junctions, or the induction of barrier cell death (necrosis/apoptosis), creating physical holes in the barrier. Although various mechanisms have already been described for *C. albicans* in different settings such as gut and oral epithelium [10, 12–14], data on crossing the epithelial cells of the BCSFB by *Candida* spp. are lacking.

We therefore investigated the mechanisms and effects of transmigration of *C. albicans* in a human in vitro model of the BCSFB.

## Methods

### Preparation of fungi

*Candida albicans* (ATCC 90028) were grown on Sabouraud glucose agar plates (BD Bioscience, San Jose, CA, USA) at 37 °C for two to three days. Yeasts were harvested by gently scraping the surface of the plates, then washed in DPBS (Gibco, Paisley, UK). The number of the yeasts was estimated in a Neubauer chamber (LO–Laboroptik, Friedrichsdorf, Germany). Yeasts were used immediately for the experiments.

### Human in vitro BCSFB model

**Fig. 1:**
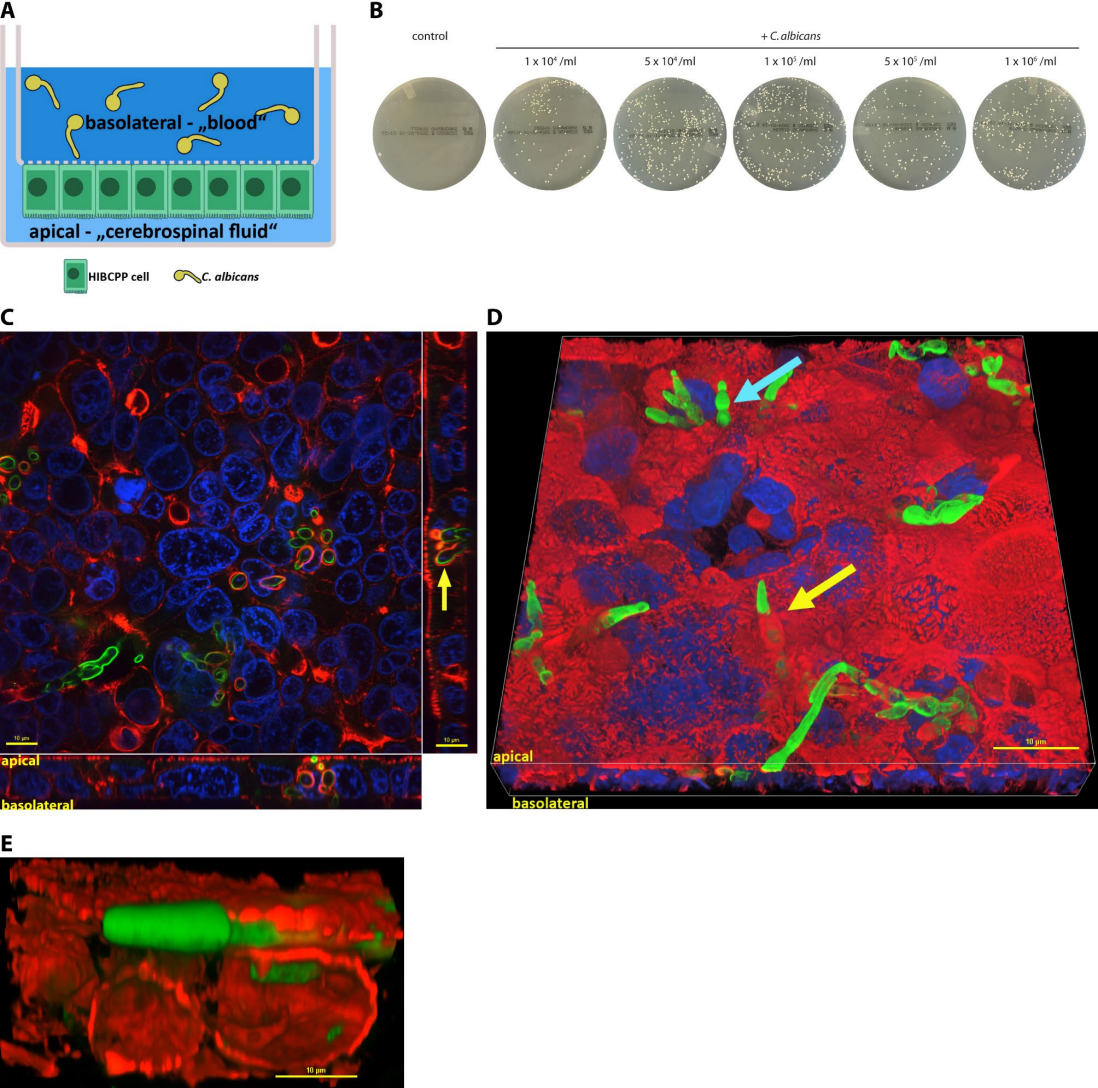
(**A**) Schematic representation of the infection model Human choroid plexus papilloma (HIBCPP) cells were grown in inverted cell cultures and infected with *Candida albicans* from the basolateral side (**B** - **E**) *Candida albicans* yeasts cross the blood cerebrospinal fluid barrier (BCSFB). *Candida albicans* was added for 24 h in various concentrations to HIBCPP cells grown in cell culture filter inserts. Uninfected HIBCPP cells served as control. (**B**) Confirmation of translocation through BCSFB by assessment of fungal growth. Culture medium from the lower compartment was harvested and seeded on Sabouraud agar plates. Shown are representative images from three independent experiments, each performed in duplicates. (**C - E**) Visualization of fungal translocation by fluorescence microscopy. HIBCPP cells were infected with 5 × 10^4^ (**D**) and 5 × 10^5^ (**C, E**) *C. albicans* yeast for 24 h. *Candida albicans* was stained green (AlexaFluor 594), cell nuclei blue (DAPI), actin red (Phalloidin 660). (**C**) Display of a cross-section through the z-plane of multiple slices in combination with a two-dimensional view from the top of the cell layer. (**D** + **E**) three dimensional depiction of Candida albicans (green) entering (**D**) and exiting (**E**) the epithelium. The cellular actin (red) surrounds the fungal hyphae (green) (yellow arrows in C and D). (**D**) Fungal hyphae are translocating close to the cell nuclei (blue arrow). (**E**) The tip of the fungal hyphae pierces through the cellular actin as the hyphae exits the cell. Z-stack images (alpha blending) were taken from regions with infected cells using Nikon NIS Element AR 4.6 imaging software (Nikon) with a 100x/1.4 NA objective lens; Shown are representative pictures from one of three independent experiments

Human choroid plexus epithelial (HIBCPP) cells derived from a human choroid plexus papilloma were used as a human in vitro BCSFB model as described [15, 16] (Fig. 1A). In brief, HIBCPP cells were cultured in a T75 flask using HIBCPP medium (DMEM/F12 (1:1), 10% fetal bovine serum (Gibco), 15 mM HEPES (Gibco), 4 mM l-glutamine (Gibco), 1% penicillin and streptomycin (Gibco), 0.05% human insulin solution (Sigma-Aldrich, Steinheim, Germany). HIBCPP cells were used between passages 21 and 38. For the in vitro BCSFB model HIBCPP cells were seeded on non-coated inverted cell culture filter inserts (filter pore size 5.0 μm, pore density 6 × 10^5^ pores per cm^2^, growth area 0.3 cm^2^; Sarstedt, Nümbrecht, Germany) with a density of 1.0 × 10^5^ cells/insert (day 0). Transepithelial electrical resistance (TEER) was measured with a tissue voltohmmeter (Milicell© ERS-2 Epithelial Volt-Ohm Meter, Millipore, Burlington, USA). When TEER values increased above 70 Ω × cm^2^, culturing medium was changed to 1% FBS HIBCPP medium [15, 16]. When TEER values were ≥ 300 Ω × cm^2^ HIBCPP cells were used for downstream experiments.

### Assessment of barrier integrity

The barrier integrity of the HIBCPP cell layer was evaluated by means of transepithelial electrical resistance (TEER) measurement. The paracellular flux of HIBCPP cells was quantified with dextran diffusion assay using fluorescein isothiocyanate (FITC)-labeled 3- kDa dextran beads (Invitrogen, Life Technologies, Eugene, USA) as previously described [17] with some modifications. In brief, dextran was added to the basolateral compartment after 20 h of infection for 4 hrs. *C. albicans* yeasts were applied to the HIBCPP cell layer in concentrations of 1 × 10^4^, 5 × 10^4^, 1 × 10^5^, 5 × 10^5^ and 1 × 10^6^ yeasts per ml from the basolateral side for 20 h (at 37 °C, 5% CO_2_). Dextran concentration in the apical cell compartment was then evaluated with a SpectraMax ID5 (Molecular Devices, San Jose, USA) at 520 nm (excitation wavelength of 490 nm). By using a standard curve, the concentration was calculated. The relative change in dextran flux was estimated by the ratio of values from control well (HIBCPP w/o *C. albicans*) and each experimental well, respectively. Analyses were performed in duplicates and determined from at least three independent experiments.

### CFU assay

The translocation of *C. albicans* through the HIBCPP cell layer was assessed by the count of colony forming units. *C. albicans* was applied on the HIBCPP cell layer grown in cell culture filter inserts from the basolateral side (upper compartment) in concentrations of 1 × 10^4^, 5 × 10^4^, 1 × 10^5^, 5 × 10^5^ and 1 × 10^6^ yeasts per ml, respectively. After 6–24 h, 100 µl of the culture medium from the lower compartment were spread on Sabouraud glucose agar plates (BD Bioscience) and the plates were incubated at 37 °C for 24 h. Culture medium from uninfected HIBCPP cell was used as control.

### Immunofluorescence microscopy

Staining and analysis of immunofluorescence was performed as previously described for primary porcine choroid plexus epithelial cells (PCPEC) and HIBCPP cells [18, 19]. Following the experiment, cell culture filter inserts were rinsed with PBS (Gibco, ThermoFisher, Waltham, MA, USA) and subsequently fixed in 3.7% formaldehyde for 15 min at room temperature (RT). The HIBCPP cell on the membranes were permeabilized with 1% Triton-X-100 (Carl Roth, Karlsruhe, Germany) PBS at RT for 20 min. Staining was performed for tight and adherens junctions (ZO-1 and E-cadherin), the cell nuclei, the actin-cytoskeleton, and *C. albicans*. As primary antibodies polyclonal rabbit α-*Candida albicans* (Invitrogen, Rockford, USA), monoclonal mouse IgG1 α-human ZO-1 (ThermoFisher Scientific, Invitrogen, Rockford, USA), mouse IgG2aκ α-human E-Cadherin (BD Biosciences, Pharmingen, San Diego, USA) were used. For the staining of actin Alexa Fluor 660 Phalloidin (Invitrogen, Life Technologies, Eugene, USA) was used. As secondary antibodies chicken α-rabbit IgG H + L Alexa Fluor 594 or goat α-rabbit IgG H + L Alexa Fluor 647, goat α-mouse IgG2a Alexa Fluor 488, goat α-mouse IgG1 Alexa Fluor 594 (Invitrogen, Life Technologies, Eugene, USA) were used. After staining, cells were washed in PBS and mounted with anti-fade reagent (Life Technologies, Carlsbad, CA, USA). All slides were imaged on a commercial laser scanning confocal microscope (Nikon C1 with a Ti-E motorized inverted microscope; Nikon, Tokyo, Japan) and analyzed using Nikon NIS Element AR 4.6 imaging software (Nikon).

### Assessment of cell viability

The assessment of cell damage of HIBCPP cells after *C. albicans* infection was performed via LIVE/DEAD^®^ Viability/Cytotoxicity Assay Kit (Invitrogen, Life Technologies, Eugene, USA) according to the manufacturer’ instructions. At the end of the incubation time, HIBCPP cells were rinsed with PBS and analyzed using Nikon NIS Element AR 4.6 imaging software (Nikon) with a 10x/1.4 NA objective lens. Excitation and emission wavelength of calcein AM were 494 nm and 517 nm, and for ethidium homodimer-1 528 nm and 617 nm.

### Cytometric bead array

Supernatants from the basolateral and apical compartment of the filter inserts were collected from HIBCPP cells incubated alone or with 1 × 10^4^*C. albicans* per ml or 1 × 10^5^*C. albicans* per ml, for 6–24 h, respectively. Levels of MCP-1 (CCL2), MIP-1α (CCL3), RANTES (CCL5), IL-8 (CXCL8), IP-10 (CXCL10), GM-CSF, IL-2, IL-10, TNF-α, IFN-γ, I-TAC (CXCL11), Fractalkine (CX3CL1) were assessed by means of a cytometric bead array (CBA; BD Biosciences) according to the manufacturer’s instructions. Limit of detection was 10 pg/ml for MCP-1, MIP-1α, RANTES, IL-8, IP-10, GM-CSF, IL-2, IL-10, TNF-α, IFN-γ, and 40 pg/ml for I-TAC, Fractalkine.

### Statistical analyses

Data were analyzed using GraphPad Prism (version 5.04; GraphPad Software, La Jolla, CA, USA). Student´s t-test was used to compare two data sets. If not otherwise stated, normality testing was performed with D’Agostino and Pearson normality test (omnibus K2) and Two-way ANOVA was used to compare multiple groups of data sets. A two-sided *P* value of less than 0.05 was considered to be statistically significant. Data are shown as mean ± SEM.

## Results

To investigate the translocation of *C. albicans* across the BCSFB, we established an in vitro cell culture filter insert assay using the HIBCPP cell line [16, 19, 20]. *C. albicans* invasion from the basolateral (= blood side) to the apical cell side (= cerebrospinal fluid (CSF) side) was investigated with a special focus on the quality of the barrier integrity and the process of fungal translocation (Fig. 1A).

### *Candida albicans* is able to cross the BCSFB

In order to explore whether *C. albicans* is able to cross the BCSFB, different concentrations of yeasts were applied to the basolateral side of HIBCPP cells. After 24 h, culture medium was taken from the apical side, and *C. albicans* colony forming units (CFU) were qualitatively assessed. Yeast growth was detected, indicating that the fungus had migrated from the upper (basolateral) to the lower (apical) compartment (Fig. 1B).

To investigate the 1mechanism of *C. albicans* translocation across HIBCPP cells, we next performed immunofluorescence analysis with a focus on the actin cytoskeleton.

*C. albicans* was detected with localized actin recruitment at the site of *C. albicans* invasion (Fig. 1C, D and E.). The penetration of HIBCPP cells by *C. albicans* hyphae was accompanied by visible formation of a cell membrane bulge around the cell penetrating hyphae (Fig. 1D, yellow arrow). Cellular actin forms a tunnel-like structure around the penetrating fungus until the hyphae exits the cell (Fig. 1E).

### Translocation of *C. albicans* across the BCSFB occurs with or without changes of the barrier integrity and impairment of tight and adherens junctions

**Fig. 2:**
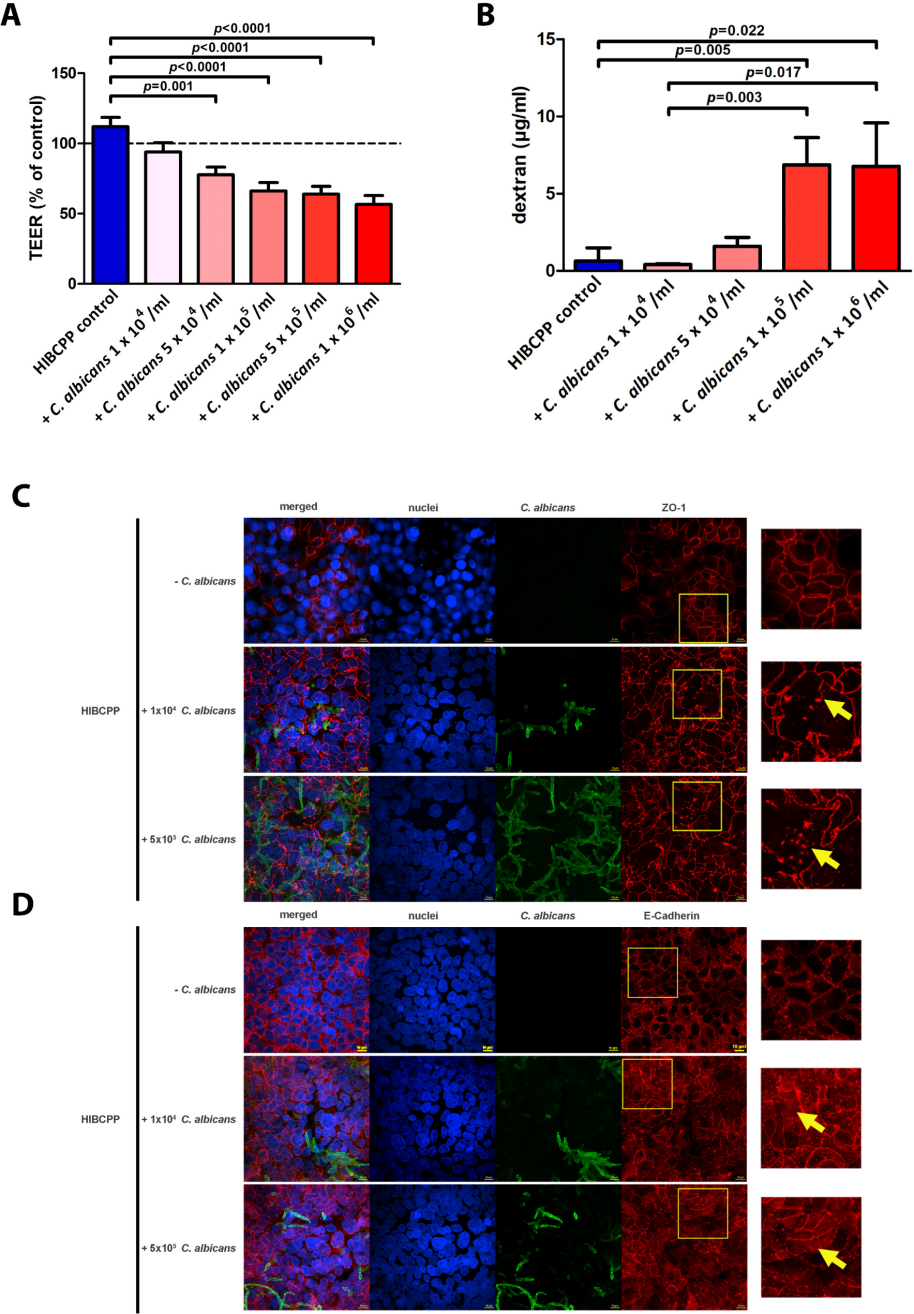
(**A** + **B**) Effect of Candida albicans on the barrier integrity of the HIBCPP cells grown in the inverted cell culture. (**A**) Increasing numbers of *Candida albicans* induce a reduction of TEER indicating loss of barrier integrity. (**B**) Dextran-flux significantly increases with concentrations of *Candida albicans* above 5 × 10^4^ per ml *Candida albicans* was added to HIBCPP cells from the basolateral side. Shown are relative values for transepithelial resistance (TEER) (**A**) and the paracellular permeability via measurement of dextran flux (**B**). HIBCPP cells incubated without fungus served as control. Columns represent relative mean ± SEM from at least 10 experiments. One-way ANOVA (TEER) or unpaired t-test (paracellular permeability) was used to analyze for significance. (**C** + **D**) Disruption of HIBCPP tight junction protein ZO-1 and the adherens junction protein E-Cadherin following infection with *Candida albicans*. The honeycomb pattern of ZO-1 is locally disrupted following *Candida albicans* infection (yellow arrows) (**C**). E-cadherin localization shifts from cell membrane to cytoplasm (**b**). The honeycomb pattern of E-cadherin is locally blurred following Candida albicans infection (yellow arrows). HIBCPP cells were infected with 5 × 10^5^*Candida albicans* yeast for 24 h. Two-dimensional images were taken from regions with infected cells using Nikon NIS Element AR 4.6 imaging software (Nikon) with a 100x/1.4 NA objective lens; On the right side, 400× enlarged images of a representative area (framed on the left) are demonstrated. Five images per condition are shown: overlay of all colors (merged), cell nuclei (blue), *Candida albicans* (green), junctions (red) and enlarged picture of the junctions (red). Shown are representative pictures from one of at least five independent experiments

The barrier function can be characterized by two major parameters, namely by TEER, which corresponds to the tightness of the cellular layer for ion flow, and by dextran flux representing the macromolecular permeability as a direct indicator of the solute flux across the barrier. Compared to the control, *C. albicans* significantly decreased TEER over 24 h, with higher fungal concentrations resulting in lower TEER (Fig. 2A). Although translocation across the BCSFB can be observed at a *C. albicans* concentration of 1 × 10^4^ yeasts per ml (Fig. 1B), no significant effect on barrier integrity was seen at this fungal concentration (93.9% ± 6.6%; mean ± SEM). Compared to the control, *C. albicans* concentrations of 5 × 10^4^, 1 × 10^5^, and 5 × 10^5^, 1 × 10^6^ yeasts per ml decreased the TEER as follows (mean ± SEM): 77.6% ± 5.5%; *p* = 0.0010, 66% ± 6%, *p* < 0.001, 64% ± 5.4%, *p* < 0.0001), and 56.6% ± 6.3%; *p* < 0.0001, respectively). For a shorter incubation of 6 hrs, no significant alterations of TEER at C. albicans concentration of 1 × 10^4^ to 1 × 10^6^ yeasts per ml were observed, whereas at incubation periods of 48 h, TEER decreased for all concentrations (data not shown).

Infection with 1 × 10^4^ yeasts per ml did not lead to an effect on dextran permeability compared to the uninfected control (Fig. 2B), indicating no effect on barrier function consistent with the results obtained by TEER measurement. Infection with *C. albicans* concentrations above 1 × 10^5^ yeasts per ml resulted in a significant increase in the paracellular permeability compared to the uninfected control (Fig. 2B).

Corroborating the results by the assessment of dextran permeability, immunofluorescence revealed that infection with *C. albicans* for 24 h resulted in the disruption of the tight junction protein ZO-1, which normally forms a pattern of a continuous honeycomb-like structure (Fig. 2C). This effect was seen for *C. albicans* concentrations of 1 × 10^4^ per ml and higher. Similar results were obtained by experiments on adherens junctions and E-cadherin, in which *C. albicans* led to re-localization of E-cadherin within HIBCPP cells (Fig. 2D, yellow arrow).

### BCSFB dysfunction can only partly be explained by *C. albicans* induced cell death

**Fig. 3:**
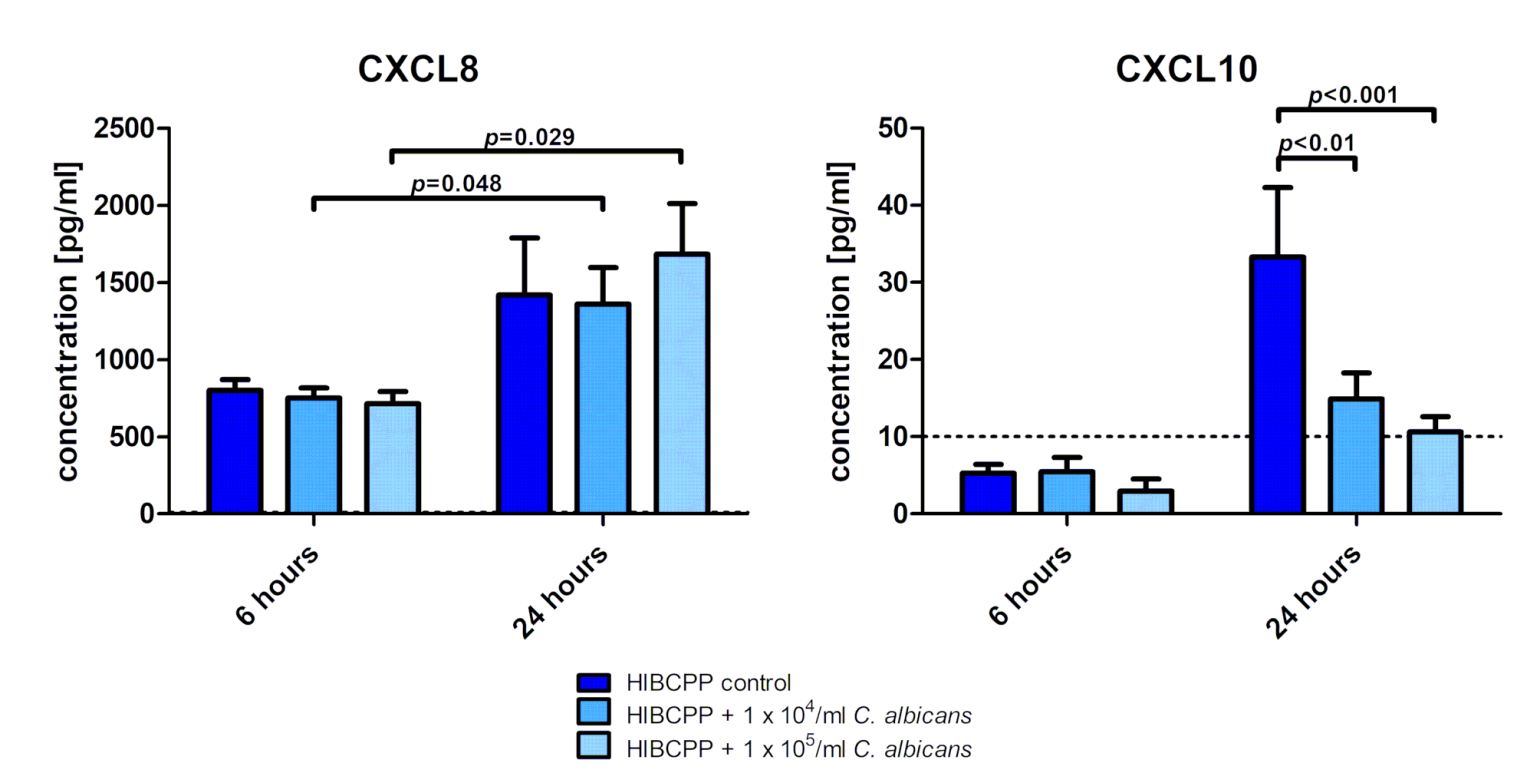
Cytokine and chemokine release by HIBCPP cells in response to *Candida albicans* infection. Supernatants were collected from the basolateral compartment and analyzed with a cytokine bead array (CBA) HIBCPP cells were infected with 1 × 10^4^*Candida albicans* per ml or 1 × 10^5^*Candida albicans* per ml, for 6–24 h, respectively. Uninfected HIBCPP cells served as control. Dotted line indicates the assay limit of detection (10 pg/ml). Shown mean ± SEM from four independent experiments

In order to assess whether *C. albicans* impairs barrier integrity by affecting HIBCPP cell viability, we infected HIBCPP cells from the basolateral cell side for 24 h with different *C. albicans* concentrations. Only minor cytotoxic effects were observed for *C. albicans* concentrations up to 5 × 10^4^/ml. In contrast to the TEER experiments in which we observed a significant effect on barrier integrity at fungal concentrations of 5 × 10^4^/ml, only *C. albicans* concentrations above 1 × 10^5^/ml had a clear cytotoxic effect in a dose-dependent manner (Fig. 3). At a concentration of 1 × 10^6^/ml, *C. albicans* led to massive cell death and disruption of HIBCPP cells (Additional file 1). Thus, our data show that the impairment of the BCSFB can only be explained in part by cell death. A similar pattern of *Candida* translocation through the HIBCPP cell layer was observed to a lesser extent 6 hrs after *Candida* infection as well as at lower concentrations of *C. albicans* (data not shown).

### *C. albicans* hyphae penetrate the BCSFB without increasing the secretion of Proinflammatory cytokines compared to uninfected controls

As pro-inflammatory cytokines are often main drivers of barrier alteration [21–24], we assessed whether infection with *C. albicans* leads to changes in the secretion of pro-inflammatory cytokines and chemokines. We assessed the cytokine secretion at both sides of the epithelium as our BCSFB model is polarized and allows the distinction of basolateral (= blood side) and apical (= CSF side) compartments. We observed a polarized secretion of cytokines to the basolateral side of the cells (Fig. 3; Additional Table 1). For CXCL8 after 24 h levels of CXCL8 did not differ significantly on the apical cell side. However, after 6 h we observed a significant increase of CXCL8 secretion to the apical cell side when HIBCPP cells were infected with 1 × 10^54^ /1 × 10^5^*C. albicans* per ml (116.9 ± 13.0 / 210.3 ± 21.1 pg/ml compared to the uninfected control (99.2 ± 9.9 pg/ml; *p* = 0.0093/ *p* = 0.0031; additional Table 1). For the basolateral cell side, after 24 h we observed both for the uninfected control and the *C. albicans* HIBCPP cells a significant increase of CXCL8 reaching comparable levels of CXCL8 for all three conditions compared to 6 h (Fig. 3 and additional Table 1). Consequently overall, infection with *C. albicans* had no significant effect on CXCL8 secretion compared to the uninfected control.

For CXCL10, we also detected an increase after 24 h for all three conditions (uninfected, 1 × 10^4^ and 1 × 10^5^*C. albicans)* compared to 6 h. CXCL10 secretion at 24 h after infection with 1 × 10^4^ and 1 × 10^5^*C. albicans* was significantly reduced to 14.9 ± 3.4 pg/ml (*p* < 0.01) and 10 ± 2 pg/ml (*p* < 0.001) when compared to the uninfected control (Fig. 3). For CCL2 and CCL5 similar effects as for CXCL10 were observed, although these effects were not significant across different durations of incubation or different concentrations of *C. albicans*, respectively (additional file 3).

No release of CCL-3 (MIP-1α), CXCL3 (fractalkine), CXCL-11 (I-TAC), GM-CSF, IL-2, IL-10, TNF-a or IFN-γ was detected to the basolateral or apical side of the HIBCPP in the presence or absence of *C. albicans* after 6–24 h, respectively (data not shown).

## Discussion

By means of a well characterized in vitro model of the BCSFB which has been repeatedly shown to have a strong barrier consisting of HIBCPP cells [15, 16, 18–20], we found that *C. albicans* hyphae are able to cross the BCSFB using strategies as the damage of barrier cells and the impairment of connecting junctions. Still, transcellular translocation seems to be the dominant pathway. These processes correlated with the fungal concentration. This in vitro model of the BCSFB has already been used for experiments with bacteria and viruses [18, 19, 25, 26], this is the first time that the translocation of fungi has been assessed.

Our results show that translocation of *C. albicans* through HIBCPP cells occurs already at low concentrations, e.g. at 1 × 10^4^/ml, which is lower than that demonstrated in comparable studies using epithelial cells of the gastrointestinal barrier [10]. Importantly, at low concentrations of *C. albicans*, translocation of the fungus across the BCSFB did not significantly affect the barrier integrity, which is in line with the findings of an in vitro model of the gut epithelial barrier [10]. Higher concentrations of *C. albicans* (e.g., more than 5 × 10^4^/ml) resulted in death of HIBCPP cells, a phenomenon which has also been observed for invasion events of *Candida* through enterocytes (Caco-2 cells) at the level of the gut epithelial barrier [10]. The peptide toxin candidalysin, encoded by the *ECE1* gene [27], has been reported to be involved in the damage of enterocytes making an efficient translocation of *C. albicans* possible [28]. Our data clearly demonstrate that translocation of *C. albicans* through the BCSFB into the brain can occur without significant changes in barrier integrity or cell damage, indicating that both, loss of integrity and cellular damage are not essential. On the other hand, our data show at the same time that the barrier integrity was already affected at *C. albicans* concentrations which do not result in significant cell death.

Our results also reveal that *C. albicans* can lead to the disintegration of the HIBCPP cell layer, which is visible by the re-localization of the tight junction protein ZO-1 and of the adhesion junction protein E-cadherin from cell membrane to the cytoplasm. However, these events occurred only at medium and high *Candida* concentrations, whereas at low concentrations, the localization of ZO-1 or E-cadherin did not change. Although we saw changes in the cell-cell connections between the HIBCPP cells, we could not detect paracellular invasion of *C. albicans* hyphae in our setting, which however does not exclude it. In contrast, although paracellular migration in the presence of altered tight and adherens junctions appears to be the easiest way of translocation, we observed that *C. albicans* translocated the BCSF barrier transcellularly via active penetration. The direct penetration of the HIBCPP cells by the fungus involved the engulfment of *C. albicans* hyphae by cellular actin, and HIBCPP cells formed a tunnel like structure, which allowed *C. albicans* hyphae to pass through the cell without destructing the cell. Similar observations with penetration as the major route for invasion by *C. albicans* have been made in in vitro models with oral and intestinal epithelial cells [10, 14]. In this respect, adhesion of *C. albicans* to oral epithelial cells triggered induced endocytosis via binding of the fungal adhesin Als3 to host E-cadherin [14, 29] and penetration was accompanied by actin surrounding the yeast [13, 14]. The HIBCPP cell actin accumulation around the *C. albicans* hyphae we observed resembles the process of induced endocytosis during infection of oral epithelial cells in vitro with *C. albicans* live yeasts [14]. In vitro models of the gut epithelial barrier demonstrated that tight and adherens junction proteins also play a role in fungal translocation [28]. It is surprising that in our model the fungus rather penetrates actively through the cells than paracellularly although this would be facilitated by the impaired junctions. This phenomenon has been described for T lymphocytes at the BBB and the BCSFB in the context of inflammation and infection with Echovirus 30, which also may become an interesting antifungal strategy. There, the route of T-cell diapedesis across the BBB was independent of loss of BBB barrier properties, but was rather regulated by the presence of endothelial ICAM-1 [30]. Infection of HIBCPP cells with Echovirus 30 led to a disruption of tight and adherens junctions, but despite E-30-induced barrier alterations leukocyte trafficking did not exclusively occur via the paracellular route [18].

Epithelial cells monitor colonization and invasion of commensals such as *C. albicans* [31], and cytokines and chemokines play a key role in immune cell migration during inflammation and fungal infection [32, 33]. In line with previous finding following infection with E-30, we detected a polarized baseline cytokine secretion to the basolateral cell side [24, 25]. Surprisingly, compared to uninfected HIBCPP cells, infection with *C. albicans* did not alter the secretion of CXCL8, CCL2, and CCL5 after 24 h, respectively, which was the fact for both the basolateral (blood) and apical (CSF) side. Similar observations were made in this model when HIBCPP cells were infected with the virus E-30 [34], whereas co-incubation with *N. meningitidis* resulted in an enhanced secretion of CXCL1-3, IL-6, CXCL8, TNF-α, G-CSF and GM-CSF, respectively [25]. The reason for this discrepancy, however, is unclear.

Our data show significantly reduced secretion of CXCL10, which has also been observed with human keratinocytes infected by *Candida* and is thought to be mediated by *Candida* prostaglandin (PG) E_2_ and is independent of cell-to-fungi contact (NHEK cells) [35]. Whether the down-regulation of CXCL10 by *Candida* favors the fungal infection as suggested in hepatitis C or Leishmaniosis [36, 37] needs to be explored.

We recognize that the results from our in vitro model may have important limitations. For example, our model does not include antifungal effector cells such as neutrophils of NK cells, and the concentrations of *C. albicans* may not reflect the *Candida* burden in the blood. Therefore, our model may not fully explain the situation *in vivo.* However, our model is unique, and the results suggest that *Candida* translocation through the BCSB will be difficult to prevent. Therefore, our next step will address the translocation of immune cells such as lymphocyte subsets or NK cells with *C. albicans* infected BCSFB, which could help to develop cellular immunotherapies to prevent or to treat CNS-infection with *C. albicans*.

## Conclusion

Taken together, our human in vitro model shows how *Candida albicans* hyphae translocate across the BSCFB. Although *C. albicans* induces barrier and cell damage at higher concentrations, the pathogen is able to cross the BCSFB in a stealthy manner without influencing barrier integrity, without inducing host cell damage or the release of pro-inflammatory cytokines. For translocation, *C. albicans* forms a tunnel-like structure, which allows the fungus the transcellular passage of the cells. As the pathogen is able to bypass the BCSFB in various ways, our data suggest that efforts to prevent CNS infection by cellular antifungal immunotherapy, e.g., with adoptively administered effector cells, seem more promising than to strengthen the BCSFB, e.g., with cytokines.

## Electronic supplementary material

Below is the link to the electronic supplementary material.

Supplementary Material 1

## Data Availability

No datasets were generated or analysed during the current study.

## Abbreviations

AJ: Adherens junction
BBB: Blood–brain barrier
BCSFB: Blood–cerebrospinal fluid barrier
BSA: Bovin serum albumin
CFU: Colony forming units
CNS: Central nervous system
CSF: Cerebrospinal fluid
DAPI: 4′-6-diamidino-2-phenylindole dihydrochloride
DMSO: Dimethylsulfoxide
FCS: Fetal calf serum, HIBCPP: Human choroid plexus papilloma cells
Hr: Hour
RT: Room temperature
SD: Standard deviation
SEM: Standard error of the Mean
TEER: Transepithelial electrical resistance
TJ: Tight junction
ZO: Zonula occludens
