# Supplementary material for: Hyphal penetration is the major pathway of translocation of Candida albicans across the blood-cerebrospinal fluid barrier
Source: Fluids Barriers CNS. 2025 Apr 4;22:34. doi: 10.1186/s12987-025-00644-x (PMC11969880; doi:10.1186/s12987-025-00644-x)
Supplement: Supplementary file 1 — Supplementary Material 1 [file 12987_2025_644_MOESM1_ESM.docx]

**Supplementary Materials**

**
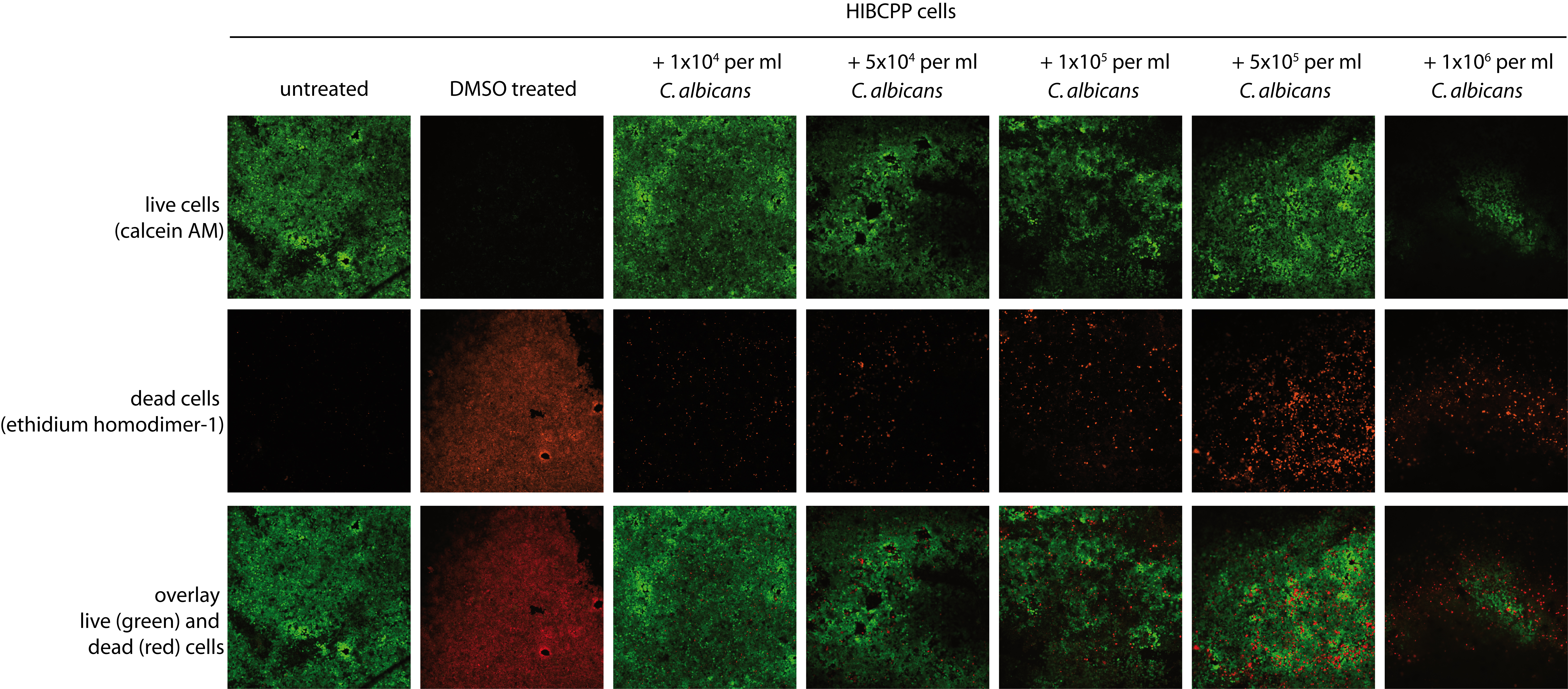
 Additional file 1:  Live/dead analysis after 24 hours of infection** The cytotoxic effect of *Candida albicans* infection of HIBCPP cells over 24 hrs is dose dependent and can be observed at fungal concentrations above 1x10^5^ per ml. Images of uninfected HIBCPP cells and HIBCPP cells infected with different amounts of Candida albicans yeasts for 24 hrs at concentrations indicated. Living cells are stained green (Calcein AM) and the dead cells are stained red (ethidium homodimer-1) using a LIVE/DEAD® viability assay. As positive staining control for ethidium homodimer-1, uninfected HIBCPP cells were treated with dimethylsulfoxide (DMSO). The pictures show representative images from three independent experiments; analyzed using Nikon NIS Element AR 4.6 imaging software (Nikon) with a 10x/1.4 NA objective lens

|  |  | **Incubation time** | | | |
| --- | --- | --- | --- | --- | --- |
| **Cytokine** |  | **6 hrs** | | **24 hrs** | |
|  |  | **basolateral** | **apical** | **basolateral** | **apical** |
| **CXCL8** | **HIBCPP control** | 800.4 ± 69.1 | 99.2 ± 9.9 | 1418.2 ± 369.4 | 309.5 ± 114.9 |
|  | **HIBCPP + *C. albicans* 1x10^4^/ml** | 751.3 ± 63.5 | **116.9 ± 13.0*** | 1359.4 ± 236.6 | 372.0 ± 86.0 |
|  | **HIBCPP + *C. albicans* 1x10^5^/ml** | 714.5 ± 77.3 | **210.3 ± 21.1**** | 1682.7 ± 329.0 | 336.7 ± 62.0 |
| **CXCL10** | **HIBCPP control** | 5.3 ± 1.1 | 0 ± 0 | 33.3 ± 9.1 | 3.2 ± 2.1 |
|  | **HIBCPP + *C. albicans*, 1x10^4^/ml** | 5.4 ± 1.9 | 0.1 ± 0.1 | **14.9 ± 3.4***** | 1.2 ± 0.8 |
|  | **HIBCPP + *C. albicans*, 1x10^5^/ml** | 2.9 ± 1.6 | 0 ± 0 | **11 ± 2****** | 0 ± 0 |
| **CCL2** | **HIBCPP control** | 18.3 ± 9.6 | 2.9 ± 1.7 | 29 ± 14.9 | 5.3 ± 1.5 |
|  | **HIBCPP + *C. albicans*, 1x10^4^/ml** | 15.4 ± 9.2 | 3.7 ± 2.3 | 20.8 ± 8.9 | 6 ± 2 |
|  | **HIBCPP + *C. albicans*, 1x10^5^/ml** | 13.8 ± 7.2 | 4.3 ± 2.2 | 21.8 ± 8.8 | 6 ± 3 |
| **CCL5** | **HIBCPP control** | 8.9 ± 2.0 | 1.6 ± 0.5 | 25.4 ± 6.8 | 4.6 ± 0.6 |
|  | **HIBCPP + *C. albicans*, 1x10^4^/ml** | 8.8 ± 1.7 | 1.6 ± 0.3 | 16.7 ± 3.7 | 4.1 ± 0.7 |
|  | **HIBCPP + *C. albicans*, 1x10^5^/ml** | 7.3 ± 1.7 | 2.2 ± 0.5 | 17.8 ± 1.7 | 2.7 ± 0.1 |

**Additional table 1: Cytokine and chemokine release by HIBCPP cells in response to *Candida albicans* infection.**

Supernatants were collected from the basolateral and apical compartment and analyzed with a cytokine bead array (CBA). HIBCPP cells were infected with 1x10^4^ *Candida albicans* per ml or 1x10^5^ *Candida albicans* per ml, for 6 or 24 hrs, respectively. Uninfected HIBCPP cells served as control. in bold are values with a significant regulation; **p*=0.009 (HIBCPP + *C. albicans*, 1x10^4^/ml vs. HIBCPP + *C. albicans*, 1x10^5^/ml); ***p*=0.003 (HIBCPP vs. HIBCPP + *C. albicans*, 1x10^5^/ml); ****p*<0.001 (HIBCPP vs. HIBCPP + *C. albicans*, 1x10^4^/ml); *****p*<0.001 (HIBCPP vs. HIBCPP + *C. albicans*, 1x10^5^/ml);
